# Polymer and small molecule mechanochemistry: closer than ever

**DOI:** 10.3762/bjoc.18.128

**Published:** 2022-09-14

**Authors:** José G Hernández

**Affiliations:** 1 Grupo Ciencia de los Materiales, Instituto de Química, Facultad de Ciencias Exactas y Naturales, Universidad de Antioquia, Calle 70 No 52-21, Medellín, Colombia

**Keywords:** ball milling, mechanochemistry, mechanophore, polymer, pulsed ultrasonication

## Abstract

The formation and scission of chemical bonds facilitated by mechanical force (mechanochemistry) can be accomplished through various experimental strategies. Among them, ultrasonication of polymeric matrices and ball milling of reaction partners have become the two leading approaches to carry out polymer and small molecule mechanochemistry, respectively. Often, the methodological differences between these practical strategies seem to have created two seemingly distinct lines of thought within the field of mechanochemistry. However, in this Perspective article, the reader will encounter a series of studies in which some aspects believed to be inherently related to either polymer or small molecule mechanochemistry sometimes overlap, evidencing the connection between both approaches.

## Introduction

**Figure 1:**
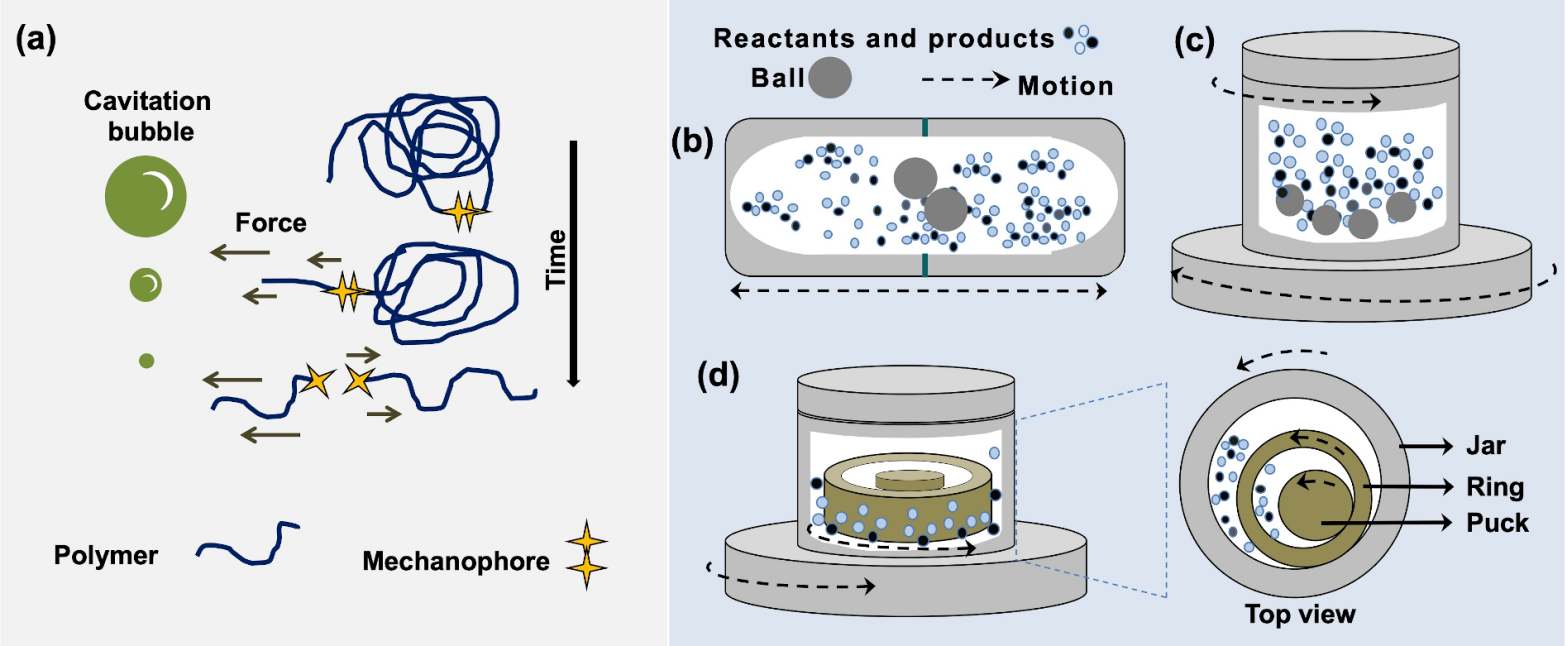
Representation of (a) cavitation and elongational flow caused by pulsed ultrasonication, (b) mixer mill, (c) planetary ball mill, and (d) ring-and-puck mill (vibrating disc mill).

In the past two decades, the growth in popularity of mechanochemistry has been unmistakable. During this time, two main experimental strategies to produce physical and chemical responses in a system when mechanical force is applied have been established. One of them, often called polymer mechanochemistry, relies on the use of polymers to transduce mechanical loads to mechanically sensitive probes (mechanophores) embedded along the polymer chains (Figure 1a) [1–4].

This is mostly accomplished through pulsed ultrasonication, and to a lesser extent by single-molecule force spectroscopy techniques [5–6]. The second approach habitually makes use of ball milling techniques to bring together small molecules (but also inorganic precursors, organometallic complexes, enzymes, monomeric units, or even polymers) in bulk and to provide the energy required for the system to react (Figure 1b and Figure 1c) [7–12]. At times, the methodological differences between both approaches seem to have created two seemingly distinct lines of thought within the field of mechanochemistry, which kept both areas to evolve mostly separately. On the one hand, polymer mechanochemistry by pulsed ultrasonication is believed to exhibit higher control at the microscopic level, for example, by enabling the transduction of mechanical cues with high directionality to the mechanophores [1–4]. In contrast, ball milling protocols have a more evident effect on the macroscopic level of the system, for instance, by causing comminution, amorphization, polymorphic transformations, structural defects, melting of the sample, etc. All these effects superimpose and influence the intrinsic reactivity of the reagents [7].

At present, both mechanochemical approaches have proven highly versatile to activate numerous chemical systems and their applicability is expected to grow. Therefore, understanding the similarities and differences between polymer and small molecule mechanochemistry has become an important subject of academic investigation and constructive discussion in review articles [13–14].

Additionally, questioning the relationship between polymer mechanochemistry and small molecule mechanochemistry has been a recurrent topic mostly addressed at scientific events. The last time I witnessed this discussion was on April 13th, 2022 at the Thieme WebCheminar #2: Mechanochemistry when Prof. Jeffrey S. Moore, the chairperson of the event, rephrased a question from one of the assistants (at 2:22:28 in the video in reference [15]). The question was initially rephrased as to what was the connection between polymer mechanochemistry and small molecule mechanochemistry. On second thoughts, Prof. Moore paraphrased the question further, ending asking if the speakers considered there was a way to use the mechanical energy in a polymeric material to ultimately have an effect on a small molecule reaction. Interestingly enough, before providing an answer, one of the participants reworded (back) the question one more time as to what was the correlation between polymer and small molecule mechanochemistry [15].

At the event, ideas on how polymer mechanochemistry could bias small molecule reactions were instantaneously provided by Prof. Stephen Craig [15], which included the possibility to mechanically control catalytic cycles in the future by switching the state of reactants or catalysts. Some approximations to this approach have already been realized. For example, some metallopolymer-based systems now enable a controllable release of metal ions by ultrasonication to trigger or catalyze small molecule reactions in solution [16–18]. Complementarily, studies on mechanically releasing cargo and unmasking organocatalytic units embedded in polymers for catalysis in small molecule systems have been discussed [15,19–20]. Moreover, the ability of force to deform the reaction energy landscape and modulate the reversibility of the reactions within proteins has been demonstrated. For example, mechanical force was used to promote the thermodynamically disfavored S_N_2 cleavage of an individual protein disulfide bond by poorly nucleophilic organic thiols [21].

On the other hand, as for the connection or correlation between polymer and small molecule mechanochemistry, this Perspective article discusses recent studies, which have found, sometimes inadvertently, links between polymer and small molecule mechanochemistry that could eventually close the apparent gap between both branches. In turn, this might reveal synergistic opportunities to strengthen the field of mechanochemistry as a whole.

## Discussion

### Activation of mechanophores in polymers by ball milling

Mechanochemical activation of mechanophores in polymeric materials is typically carried out by pulsed ultrasonication [5–6]. Under such conditions, the collapse of cavitation bubbles in the liquid medium creates a gradient that exerts mechanical force along the polymer backbone, ultimately reaching and activating the mechanophores within it (Figure 1a). The technical simplicity of the method and the compatibility with spectroscopic analytical techniques for monitoring [22] have made sonication of polymer solutions the primary method in the field of polymer mechanochemistry. However, in recent times, manual grinding and ball milling techniques (Figure 1b and Figure 1c) have also been used as alternatives to activate mechanophores incorporated in polymers. Such an application complements the original use of ball milling to generate mechanoradicals through homolytic cleavage of the polymer chains [23], a strategy that is still of relevance today [24–25] and also complements the recent application of ball milling for the synthesis of polymers [26].

**Scheme 1:**
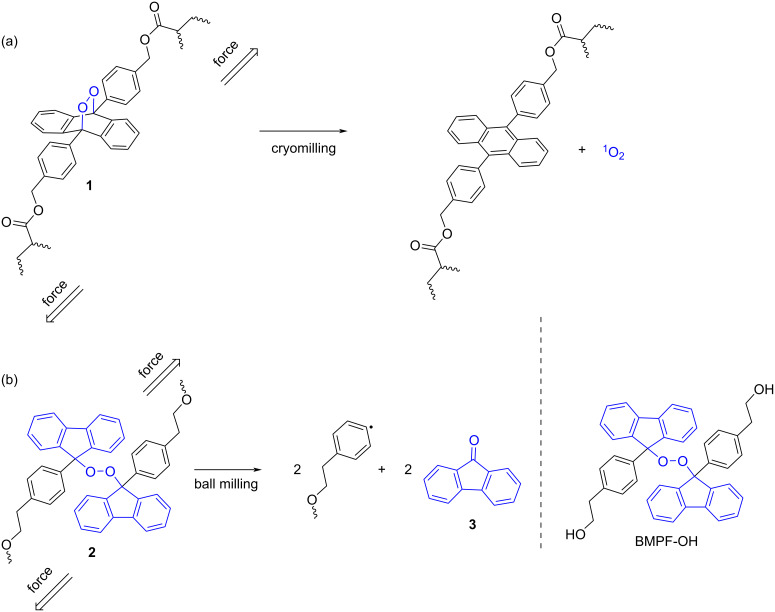
(a) Mechanochemical activation of anthracene–endoperoxide mechanophore incorporated in the cross-linked polyacrylate polymer **1** by cryomilling in a mixer mill. (b) Mechanochemical activation of a polymer network matrix, bis(9-methylphenyl-9-fluorenyl) peroxide (BMPF)-containing poly(butyl methacrylate) **2**, in a mixer mill.

As an example for the use of ball milling to activate mechanophores incorporated in polymers, Baytekin, Akkaya, and co-workers found that ball milling of the cross-linked polyacrylate polymer **1** could trigger the release of singlet oxygen from the anthracene–endoperoxide mechanophores (Scheme 1a) [27].

To support the claim that the generation of ^1^O_2_ occurred mechanically rather than thermally due to local heat formation by ball collision, the authors tested the mechanochemical reaction under cryogenic ball milling conditions and found that even at low temperature, the mechanical treatment was enough to facilitate the cycloreversion process (Scheme 1a) [27].

In a related work, in 2021, Otsuka and co-workers incorporated a BMPF mechanophore into glassy and rubbery polymeric networks such as poly(butyl methacrylate) and a poly(hexyl methacrylate) [28]. Upon treatment of the polymeric material **2** in a mixer mill (Figure 1b), the BMPF units underwent a mechanical homolytic fragmentation of the O−O peroxide bond, releasing fluorescent 9-fluorenone (**3**) via β-scission (Scheme 1b). To highlight the importance of the polymer network in the transduction of the mechanical stimulus to the mechanophore, the authors demonstrated that ball milling monomeric BMPF-OH at 30 Hz for 3 h only generated traces of **3** (1% after isolation). Importantly, the BMPF mechanophore was also proven to be thermally stable up to 110 °C, which supports the conclusion that the mechanical force caused by the grinding was responsible for the activation of the BMPF mechanophores [28].

**Scheme 2:**
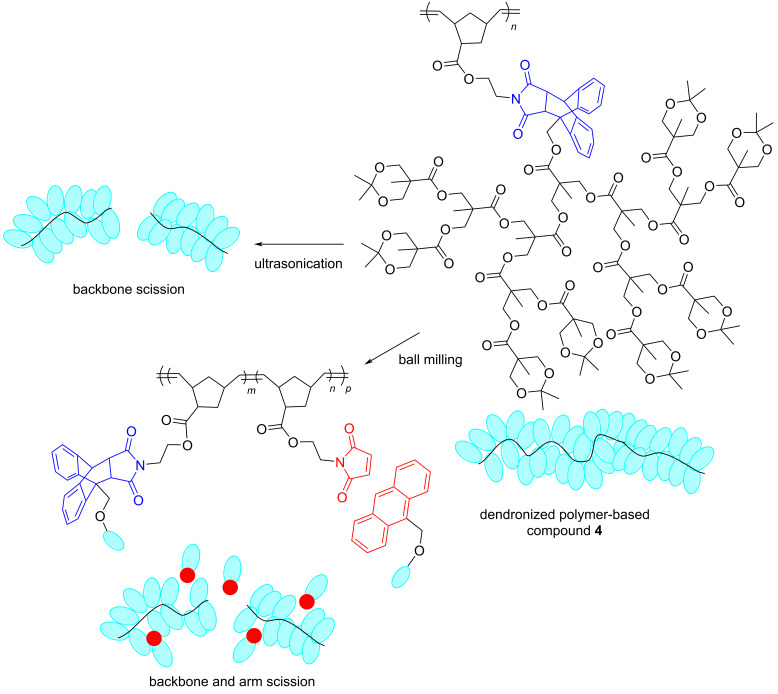
Mechanochemical activation of dendronized polymer-based compound **4** by ultrasonication and ball milling in a mixer mill.

Comparative studies between ultrasound and ball milling have also demonstrated that both experimental approaches can transduce mechanical force to polymeric materials, sometimes in a complementary manner. For example, in 2021, Noh, Peterson, and Choi investigated the mechanochemical degradation of bottlebrush and dendronized polymers when exposed to ultrasonication in solution and when ball milled in the solid state [29]. The results for bottlebrush polymers demonstrated a more pronounced arm scission by ball milling than by sonication, compared to the extent of backbone rupture. However, for dendronized polymers, arm scission could be accomplished by ball milling but it was not observed in sonication experiments. These observations inspired the synthesis and activation of mechanophores (maleimide–anthracene cycloadducts) in dendronized polymer-based materials such as **4** upon ball milling (Scheme 2).

The differences observed in the activation of polymers such as **4** between ball milling and ultrasonication were associated with the more restricted chain mobility in the solid state and with the dissimilar distribution of mechanical forces on the backbone and arms. With ball milling, these were more pronounced towards the arms of the polymer [29].

Other studies have also provided mounting evidence that ball milling and manual grinding, techniques typically used in small molecule mechanochemistry, could trigger chemical reactions in multimechanophore polymers [30], nonsymmetric mechanophores embedded in polymer systems [31], and mixtures of mechanochromic polymers [32], among others [33–34]. Therefore, it has been clearly demonstrated that pulsed ultrasound-based, grinding and ball milling techniques are competent in activating polymeric materials and, more importantly, that sometimes the apparent disparity between both approaches can actually lead to complementary reactivity, as discussed above.

### Depolymerization of biomacromolecules by mechanical force

**Figure 2:**
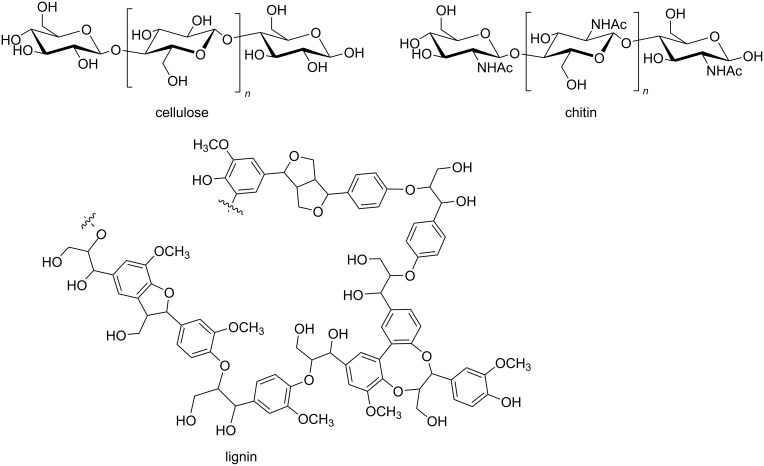
Structure of cellulose and chitin and approximation to the structure of lignin.

In addition to the manipulation of manufactured polymers, ball milling techniques have also been reported to facilitate the depolymerization of biopolymers [35]. On the one hand, the implementation of solvent-free ball milling has enabled to surpass the insolubility and recalcitrant reactivity of cellulose [36–37], chitin [38–39], and lignin (Figure 2) [40–41].

**Figure 3:**
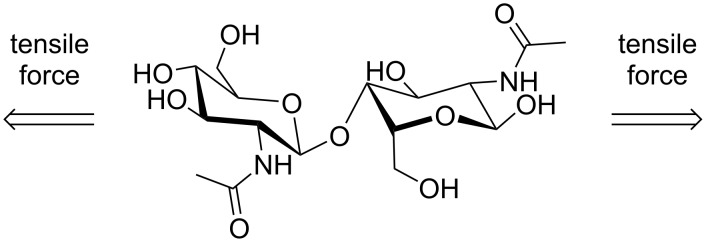
Tensile forces by ball milling change the conformation of a chitin model compound. This deformation facilitates the subsequent cleavage of glycosidic bonds to produce oxocarbenium ion intermediates for hydrolysis [45].

In addition to allowing solvent-free reactions, mechanical forces generated inside ball mills can depolymerize biomass through cleavage pathways that are different from those found in solution. Computational studies have been a key to understand the role of mechanical force in such reactions and to explain the changes in selectivity under force [42–44]. For example, experimental results have demonstrated that ball milling in a planetary ball mill (Figure 1c) enhances the depolymerization of acid-impregnated chitin more selectively towards glycosidic bond cleavage (i.e., backbone rupture) over amide bond breakage (i.e., deacetylation) [38,45]. Notably, the result is different for the reaction in solution, where scission of both bonds is observed [46]. This difference is believed to be related to the exertion of tensile forces along the glycosidic linkage of the polymer chain during ball milling, which may lower the activation energy for the depolymerization of chitin. Indeed, DFT calculations using the *N*-acetylglucosamine dimer as the model compound showed that the application of pulling forces to selected atoms in the dimer perturb the reaction, making the depolymerization easier to occur [45]. In contrast, no change in the activation energy of the deacetylation step was observed with the introduction of the pulling forces. The decrease in the activation energy for the mechanochemical depolymerization of chitin was attributed to force-induced conformational changes in the structure, which destabilize the reactant state upon the introduction of a sufficient pulling force (Figure 3).

**Figure 4:**
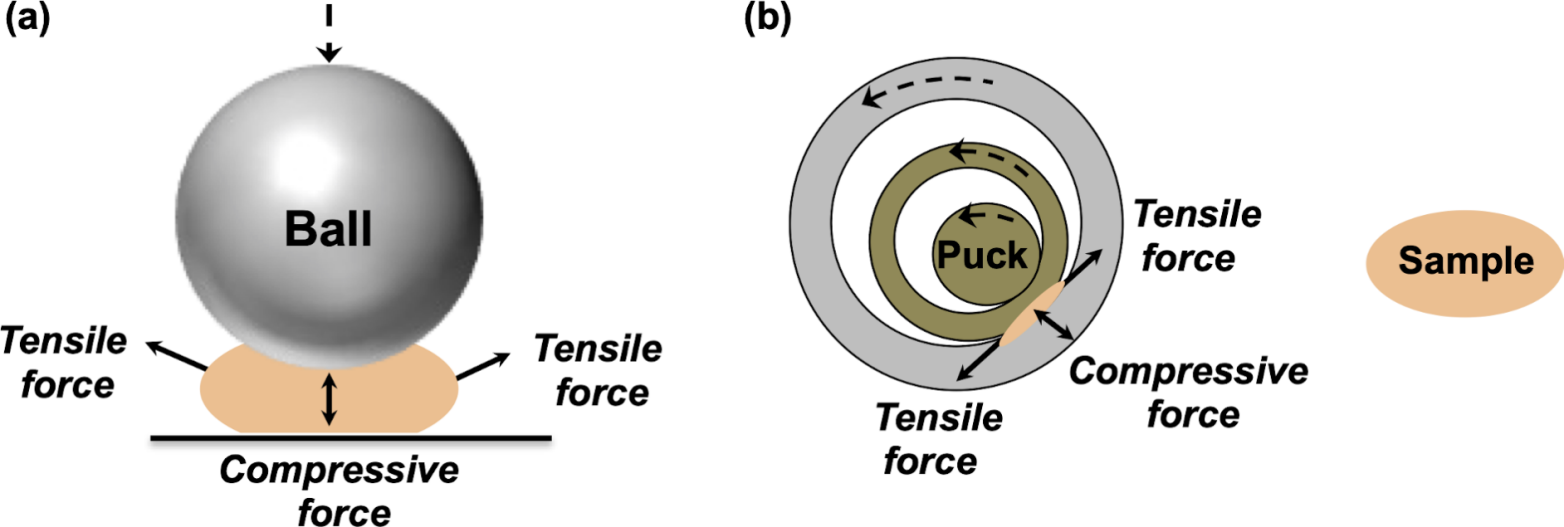
(a) Representation of a collision between the ball and a particle of a chitin sample and (b) mechanical treatment of a particle of a lignin sample in a ring-and-puck mill.

Evidently, ball milling techniques apply forces to the samples in a random fashion, with friction, shearing, and compression being more prominent than pulling forces. Well aware of this, further studies by Kobayashi, Fukuoka, and co-workers on the depolymerization of chitin by ball milling considered not only tensile but also compressive forces transduced by the impact of the balls on the milled sample (Figure 4a) [47].

The computational investigation revealed that ball milling can provide a subnano- to nanonewton order of tensile and compressive forces to activate the biopolymer. Moreover, the results corroborated that tensile force applied in the direction of the polymer chain activates the scission of the glycosidic bonds, but they also showed that the pulling force transduced by ball milling would be insufficient to impact the reaction significantly. Therefore, the depolymerization of chitin was found to be less frequently influenced by tensile forces. In contrast, compressive forces in the same direction as the collision less strongly activate the chemical bonds, but the large number of this type of collisions in a planetary ball mill (Figure 1c) can add up and provide compressive forces large enough for the activation of glycosidic bonds [47].

In the same work, Kobayashi, Fukuoka, and co-workers concluded that “if a new mechanical method [different from ball milling] is developed to apply tensile forces to samples selectively and efficiently, it will probably improve the reaction efficiency” [47]. Along these lines, ring-and-puck mills (often called vibrating disc mills, Figure 1d) are a type of milling equipment in which the number of collisions is low, but the friction, and therefore the tensile force transduced to the milled sample, is high. Experimental studies focused on the oxidative mechanochemical depolymerization of lignin have found that mechanical treatment of this biomacromolecule by ring-and-puck milling can lead to the cleavage of lignin linkages more effectively and in a shorter time as compared to the reaction carried out in ball mills [48–49]. Probably, the compression and particularly the tension exerted on the sample by the disc is transduced with better directionality to the biopolymer, which acts as a force transmission medium (Figure 4b). Hence, widespread mechanical methods such as ring-and-puck milling are already available to mechanochemically activate polymeric materials with an enhanced control over the direction in which the mechanical force is applied. However, it remains to be seen whether ring-and-puck milling, or similar technologies, will be able to trigger the mechanical activation of multimechanophore polymers. If successful, this approach could actually enable the activation of functional polymers on a scale of up to a hundred grams per run.

### Piezoelectric materials as mechanophores

**Figure 5:**
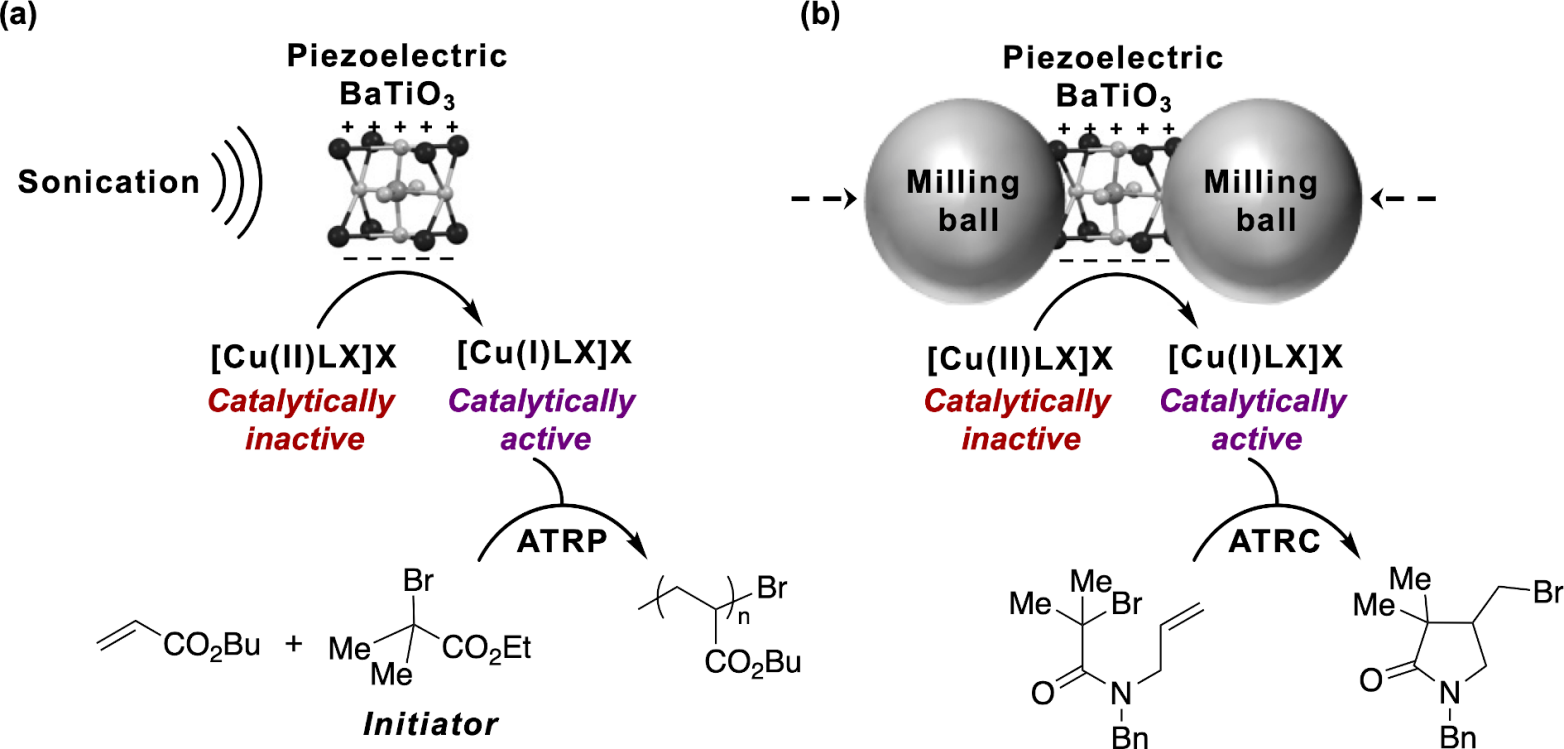
(a) Ultrasound-induced ATRP using piezoelectric BaTiO_3_ and (b) mechanochemical atom transfer radical cyclization (ATRC) using BaTiO_3_ by ball milling.

Mechanophores were initially described as molecular units that chemically respond in a selective manner to a mechanical perturbation preferentially transduced through polymers [1]. As mentioned above, force-sensitive molecular mechanophores are now complemented by organometallic-based force-responsive mechanophores that are not purely organic molecular fragments [16–18]. In fact, the concept of a mechanophore is now broader and may include any force-reactive functional unit, whether it possesses mechanically labile bonds or not, and whether it is a hydrocarbon [50], contains heteroatoms [51], or is inorganic in nature. With regards to the latter, ceramic piezoelectric materials such as barium titanate (BaTiO_3_) or zinc oxide (ZnO) are materials that can accumulate electric charge in the structure in response to applied mechanical stress, and thus they could well be considered as force-reactive functional units (i.e., a new class of mechanophores) [52]. Interestingly, techniques based on ultrasound and ball milling have recently been used to activate piezoelectric materials, hinting at the similarities of these two different experimental approaches in the development of mechanochemical reactions. For example, Li and co-workers showed that piezoelectric nanoparticles such as BaTiO_3_ and ZnO could trigger water electrolysis under ultrasonication [53]. Sonication caused deformation of, or strain on the material, which induced a nonzero dipole moment in the crystal lattice. As a consequence, a strain-induced charge potential of at least 1.23 eV was produced on the surface of the material, leading to the conversion of mechanical energy into chemical energy. Mechanistically, the ZnO and BaTiO_3_ participated in the formation of H_2_ and O_2_ from the water splitting reaction by donating strain-induced electrons and holes [53]. The piezoelectricity obtained upon ultrasonication of BaTiO_3_ has also been used to trigger and sustain atom transfer radical polymerization (ATRP) reactions of acrylate monomers by mechanoredox reduction of inactive Cu(II) salts to catalytically active Cu(I) species (Figure 5a) [54].

Complementarily, ball milling of BaTiO_3_ has been used for mechanoredox ATRC reactions in which highly polarized BaTiO_3_ particles produced by the collisions of the milling balls were proposed to reduce the Cu(II) precatalyst to the catalytically active Cu(I) form (Figure 5b) [55].

Other examples have also shown how mechanical activation of piezoelectric materials by ultrasonication and by ball milling is equally appropriate for mechanochemical reactions. For instance, in the field of environmental remediation, the degradation of dye pollutants (e.g., rhodamine) has been accomplished using BaTiO_3_ as a mechanophore in solution with ultrasonication [52] or under solvent-free ball milling reaction conditions [56]. These reports complement recent studies on piezocatalysis, such as on chain-growth polymerizations [57–59], arylations and borylations [60], trifluoromethylations [61], as well as dehydrogenative couplings and cycloadditions [62], among others [63–64] in which mechanically polarized piezoelectric materials triggered redox chemistry.

### Alternative strategies to transduce mechanical force to mechanophores

**Figure 6:**
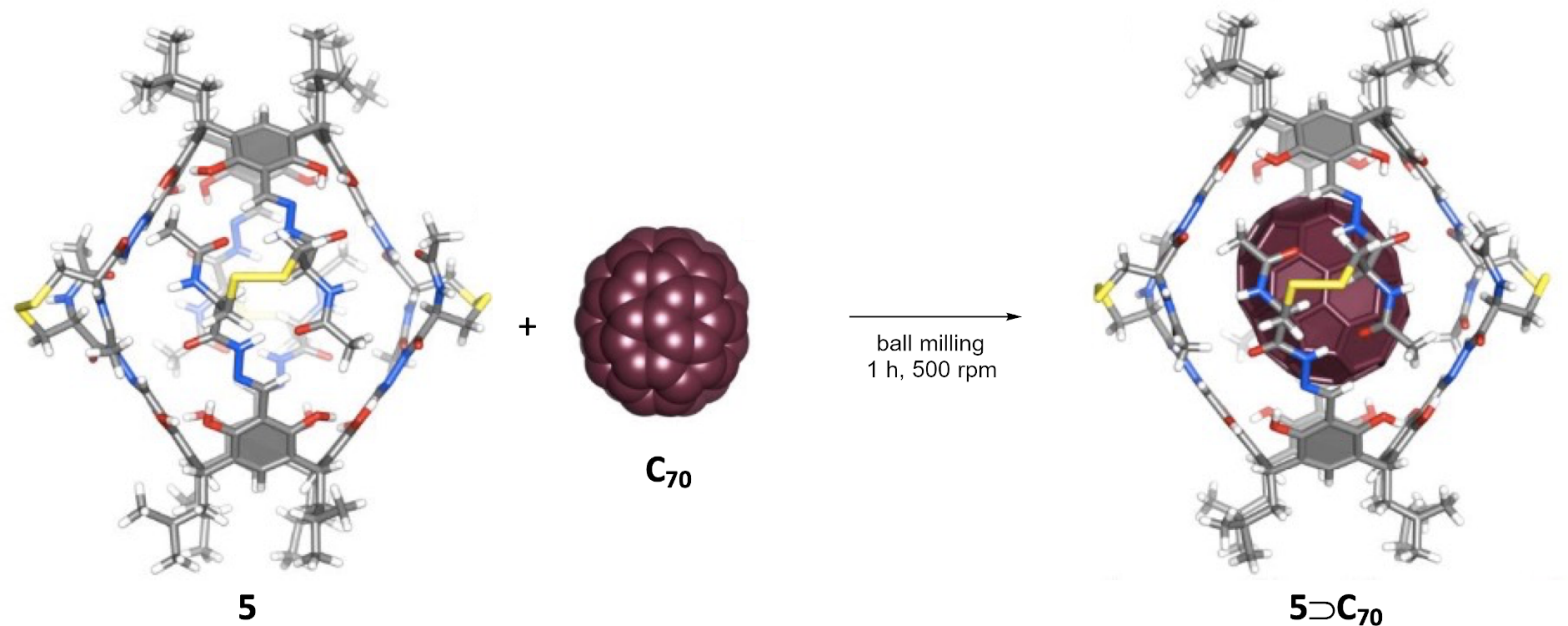
Mechanochemical solid-state complexation of organic capsule **5** with fullerenes C_70_ in a planetary ball mill. Figure 6 was adapted from [65], H. Jędrzejewska et al., “Porous Molecular Capsules as Non- Polymeric Transducers of Mechanical Forces to Mechanophores”, Chem. Eur. J., with permission from John Wiley and Sons. Copyright © 2019 WILEY‐VCH Verlag GmbH & Co. KGaA, Weinheim.

As evidenced in previous paragraphs, the concept of a mechanophore, which was originally associated with polymer mechanochemistry, has reached the field of small molecule mechanochemistry. However, not only the definition of a mechanophore has evolved in recent years but also the means to transduce mechanical energy to the mechanophores. Typically, polymers were the force transmission medium of choice, but in a recent study, Potrzebowski, Szumna, and co-workers have shown that molecular capsules can behave as stress-sensitive units [65]. The authors demonstrated the effective complexation of the covalent capsule **5** (made from resorcinarene caps connected by four dihydrazone units of ʟ-cysteine) with C_60_ and C_70_ fullerenes upon neat ball milling in a planetary ball mill (Figure 6).

The mechanochemical complexation is remarkable since the porous capsule does not possess large enough openings for the fullerenes to pass through. However, the capsule **5** is sensitive to mechanochemical stress due to the porosity, conformational rigidity, and due to the presence of hydrazone and/or disulfide moieties that act as mechanophores. Therefore, during ball milling, **5** gets partially disintegrated at the weakest covalent connections, enabling the access of fullerenes. As a result, besides polymeric matrices, also porous, semirigid molecules [65] and molecular anvils [66] could eventually become effective transducers of mechanical forces to mechanophores.

**Scheme 3:**
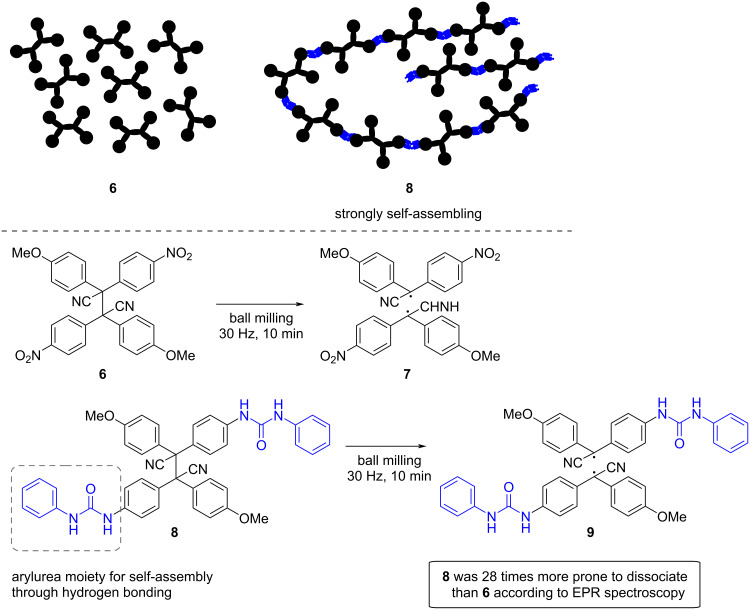
Comparative mechanochemical dissociation of the central C–C bond in TASN derivatives **6** and **8**.

Simpler strategies to circumvent the need for polymers as force transmission media have been investigated. In 2021, Otsuka and co-workers reported supramolecular hydrogen-bonding systems as alternative mechanical force transducers [67]. Specifically, the authors synthesized tetraarylsuccinonitrile (TASN) derivatives **6** and **8**. TASN is a well-known mechanophore that generates diarylacetonitrile radicals under force. Hence, when TASN derivative **8**, bearing diarylurea moieties, was ball milled, the corresponding radical **9** was detected by electron paramagnetic resonance (EPR) spectroscopy. Similar treatment proved that **6** was 28 times less prone to generate radicals (Scheme 3) [67].

The difference in the C–C bond scission between **6** and **8** was explained based on the ability of diarylurea moieties in **8** to form strong self-assemblies through hydrogen bonding. In the solid state, this enabled the transduction of mechanical force to the mechanophores. Moreover, it was demonstrated that the hydrogen bonds of the diarylurea linkages also acted as supporting units to maintain the activated mechanophores (radicals) for a longer time [67]. Overall, this new strategy, which harnesses the power of noncovalent interactions by ball milling [68–70], could become an alternative to enhance mechanochemical bond scission in mechanophores without the need to incorporate them into polymeric matrices.

## Conclusion

Ultrasonication and ball milling have historically been the flagship techniques in the fields of polymer and small molecule mechanochemistry, respectively. At the same time, examples of crossover in the literature were scarce. However, recent studies have evidenced that not only ultrasound but also ball milling can trigger the activation of mechanophores incorporated into polymeric matrices. Conversely, ultrasonication in solution has proven highly effective to mechanically polarize redox active mechanophores such as piezoelectric materials, for which the activation in solid state by ball milling is becoming more frequent. Therefore, despite the apparent differences between both methodological approaches, which is partially attributed to the ability of each method to strain chemical systems on different length- and timescales, new studies have proven that the disparity is slowly becoming less pronounced. For example, with regards to the accepted higher directionality to induce mechanical deformation at the molecular level exhibited by ultrasonication experiments, a new body of evidence indicates that tensile and compressive forces exerted by ball and ring-and-puck milling can also be transduced through the backbone of large molecules. This is accompanied by a certain degree of directionality and can therefore influence the scission of specific bonds within the material. Moreover, research at the interface of polymer and small molecule mechanochemistry has opened new avenues towards the activation of mechanophores without the need for incorporation into polymeric materials. This is particularly true for porous, semirigid capsules and molecular anvils, which hold promise to become standard low-molecular effective transducers of mechanical forces. This, together with the ability of ball milling to facilitate the formation of covalent and noncovalent supramolecular assemblies, could lead to the direct activation of small mechanophores via the formation of permanent of transient structural aggregates to simplify the transduction of mechanical force to force-reactive functional units. Looking into the future, one could expect that additional comparative studies between sonication and ball milling experiments will occur. At the same time, research into new modes to activate matter by force (e.g., twin-screw extrusion [71], resonant acoustic mixing [72], vortex fluidic mixing [73], laminar flow [74], etc.) will continue to unveil similarities and complementarities, rather than disparities, between the ways polymer and small molecule mechanochemical reactions occur. As a result, the field of mechanochemistry as a whole will ultimately be strengthened.
